# Perceived stress and non-alcoholic fatty liver disease in apparently healthy men and women

**DOI:** 10.1038/s41598-019-57036-z

**Published:** 2020-01-08

**Authors:** Danbee Kang, Di Zhao, Seungho Ryu, Eliseo Guallar, Juhee Cho, Mariana Lazo, Hocheol Shin, Yoosoo Chang, Eunju Sung

**Affiliations:** 1 Department of Clinical Research Design & Evaluation, SAIHST, Sungkyunkwan University, Seoul, Korea; 2 Center for Clinical Epidemiology, Samsung Medical Center, Sungkyunkwan University School of Medicine, Seoul, Korea; 3 Department of Epidemiology and Welch Center for Prevention, Epidemiology, and Clinical Research, Johns Hopkins University Bloomberg School of Public Health, Baltimore, Maryland United States of America; 4 Center for Cohort Studies, Total Healthcare Center, Kangbuk Samsung Hospital, Sungkyunkwan University School of Medicine, Seoul, Korea; 5 Department of Occupational and Environmental Medicine, Kangbuk Samsung Hospital, Sungkyunkwan University School of Medicine, Seoul, Korea; 6 Department of the Family Medicine, Kangbuk Samsung Hospital, Sungkyunkwan University School of Medicine, Seoul, Korea

**Keywords:** Non-alcoholic fatty liver disease, Risk factors

## Abstract

Psychological stress may have adverse metabolic effects and induce unhealthy behaviors, but the role of stress in the development of non-alcoholic fatty liver disease (NAFLD) is largely unexplored. We investigated the association between perceived stress and the prevalence of NAFLD in a large sample of apparently healthy men and women. We performed a cross-sectional study of 171,321 adults who underwent health screening examination between 2011 and 2013 in one health screening center. Perceived stress was assessed using the short version of the Perceived Stress Inventory (PSI). NAFLD was assessed using ultrasonography in the absence of excessive alcohol use or any other identifiable cause of liver disease. The prevalence of NAFLD was 27.8%. In fully-adjusted multivariable models, the odds ratio (95% confidence intervals) for NAFLD comparing participants in the 5^th^ quintile of PSI score (≥23) with those in the lowest quintile (<12) was 1.17 (1.11, 1.22), with a moderately increased prevalence of NALFD across quintiles of PSI score. The positive association between PSI score and NAFLD was observed in all subgroups analyzed, although the association was stronger in men compared to women (p interaction <0.001), and in obese compared to non-obese (p interaction 0.005). In this large study of apparently healthy men and women, higher perceived stress was independently associated with an increased prevalence of NAFLD, supporting a possible relationship between perceived stress and NAFLD. Prospective study is needed to elucidate mediating mechanisms to warrant stress management to reduce NAFLD.

## Introduction

Non-alcoholic fatty liver disease (NAFLD) is the most common chronic liver disease worldwide, with prevalence ranging from 25 to 45% of the general population^1^. NAFLD is an important public health problem because of its high prevalence, potential progression to severe liver disease, and strong link with cardiovascular risk factors and cardiovascular disease^2^. Thus, it is important to develop strategies to identify high-risk individuals before they develop NAFLD and to identify potentially modifiable risk factors^3^.

Stress is a recognized risk factor for cardiovascular and metabolic disease^4^. Stress initiates a complex spectrum of biological^5^ and behavioral reactions^6^ that result in activation of the hypothalamic-pituitary adrenocortical (HPA) axis and stimulation of the sympathetic nervous system (SNS) with increased levels of epinephrine, cortisol, and pro-inflammatory cytokines^7,8^, all of which could induce NAFLD^9^. Additionally, stress can lead to unhealthy behaviors, including alcohol intake, tobacco use, and poor diet, which are also associated with fatty liver^8,10^.

Evidence regarding the association of stress with NAFLD is very limited. A few studies have evaluated the association between biological markers of stress, such as cortisol and heart rate variability, with liver disease^11–13^ or the association of perceived stress with liver-related mortality^14^. However, no study has evaluated the association of perceived stress with NAFLD. Therefore, the aim of this study was to evaluate the association between perceived stress and NAFLD measured by ultrasound (US) in a large sample of apparently healthy men and women who participated in a health screening program.

## Results

The mean (standard deviation) age and PSI score of study participants were 39.8 (9.0) years and 16.9 (6.7), respectively (Table 1). Patients with higher PSI score were younger and more likely to be female, unmarried, highly educated, current smokers and alcohol drinkers (Supplemental Table 1). The prevalence of NAFLD was 27.8%. Participants with NAFLD were older and more likely to be male, educated, married, current smokers and alcohol drinkers, and to have higher BMI, blood pressure and cholesterol levels compared to those without NAFLD.

**Table 1:** Baseline characteristics of study participants overall and by nonalcoholic fatty liver disease (NAFLD) status^*^.

Characteristic	Overall (n = 171,321)	NAFLD		p value
		No (n = 123,783)	Yes (n = 47,538)	
PSI score	16.9 (6.7)	17.0 (6.7)	16.8 (6.6)	<0.001
Age (years)	39.8 (9.0)	38.9 (8.8)	42.0 (9.1)	<0.001
Sex				<0.001
Female	85,560 (49.9)	74,454 (60.1)	11,106 (23.4)	
Male	85,761 (50.1)	49,329 (39.9)	36,432 (76.6)	
Education				<0.001
<High school	4,757 (2.8)	3,089 (2.5)	1,668 (3.5)	
High school or technical college	49,923 (29.1)	37,651 (30.4)	12,272 (25.8)	
≥University	110,618 (64.6)	78,911 (63.7)	31,707 (66.7)	
Marital status				<0.001
Unmarried	25,493 (14.9)	20,268 (16.4)	5,225 (11.0)	
Married	139,715 (81.6)	99,349 (80.3)	40,366 (84.9)	
Separated, divorced or widowed	3,371 (2.0)	2,288 (1.8)	1,083 (2.3)	
Study center				<0.001
Seoul	106,922 (62.4)	74,410 (60.1)	32,512 (68.4)	
Suwon	64,399 (37.6)	49,373 (39.9)	15,026 (31.6)	
Smoking				<0.001
Never	94,849 (55.4)	75,319 (60.8)	19,530 (41.1)	
Former	23,729 (13.9)	14,084 (11.4)	9,645 (20.3)	
Current	29,696 (17.3)	16,896 (13.6)	12,800 (26.9)	
Alcohol				<0.001
None	29,440 (17.2)	22,916 (18.5)	6,524 (13.7)	
Moderate	126,666 (73.9)	89,433 (72.2)	37,233 (78.3)	
Vigorous exercise (times/week)				<0.001
0	103,059 (60.2)	75,188 (60.7)	27,871 (58.6)	
1–3	51,328 (30.0)	35,950 (29.0)	15,378 (32.3)	
>3	11,412 (6.7)	8,718 (7.0)	2,694 (5.7)	
Body mass index (kg/m^2^)	23.0 (3.3)	21.9 (2.7)	25.9 (3.0)	<0.001
Total cholesterol (mg/dL)	193.9 (34.1)	189.0 (32.1)	206.6 (35.8)	<0.001
HDL cholesterol (mg/dL)	58.2 (14.7)	61.7 (14.4)	49.0 (11.2)	<0.001
Triglycerides (mg/dL)^†^	87.0 (62.0–129.0)	76.0 (57.0–105.0)	135.0 (96.0–188.0)	<0.001
Systolic blood pressure (mmHg)	108.0 (13.2)	105.4 (12.5)	114.9 (12.5)	<0.001
Hypertension	17,406 (10.2)	8,166 (6.6)	9,240 (19.4)	<0.001
Diabetes	5,685 (3.3)	1,858 (1.5)	3,827 (8.1)	<0.001

^*^Values are means (standard deviation) or number (%).

^†^Values are median (interquartile range).

PSI = Perceive Stress Inventory; HDL = high-density lipoprotein.

**Figure 1:**
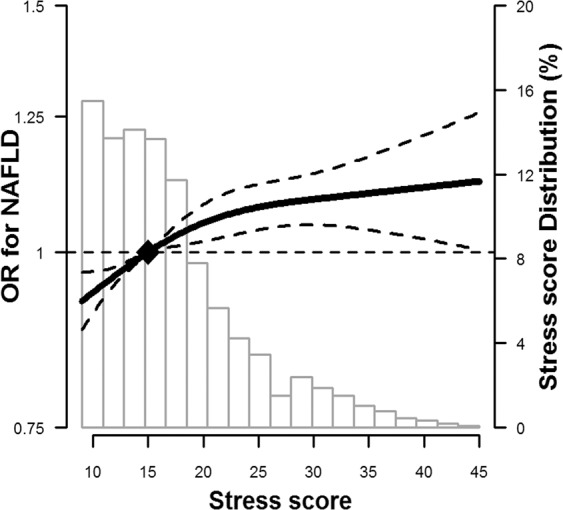
Flowchart of study participants.

Age and sex adjusted prevalence of NAFLD were 26.7%, 27.1%, 27.5%, 28.3% and 29.5% in 1st, 2nd, 3rd, 4th and 5th quintile of PSI score, respectively (data not shown). In fully-adjusted multivariable models, the OR (95% CI) for NAFLD comparing participants in the 5th quintile of PSI score (≥23) with those in the lowest quintile (<12) was 1.17 (1.11, 1.22), with a moderately increased prevalence of NAFLD across quintiles of PSI score (p trend <0.001; Table 2). In spline regression models, the association between PSI score and the prevalence of NAFLD was nonlinear, with a stronger association at low levels of PSI than at high levels (p value for nonlinear spline terms = 0.04; Fig. 1). The OR (95% CI) comparing participants in the 90^th^ percentile to those in the 10^th^ percentile of PSI scores in the spline regression model was 1.16 (1.11, 1.20). Adjusting for multiple confounders and possible mediators did not substantially affect the results.

**Table 2:** Adjusted odds ratios (95% CI) for non-alcoholic fatty liver disease (NAFLD) by quintile of perceive stress inventory (PSI) score (n = 171,321).

	Model 1^*^	Model 2^†^	Model 3^‡^
PSI score quintile (range)			
Q1 (9–11)	1.00 (reference)	1.00 (reference)	1.00 (reference)
Q2 (12–14)	1.02 (0.98, 1.05)	1.01 (0.98, 1.05)	1.05 (1.00, 1.09)
Q3 (15–17)	1.04 (1.01, 1.08)	1.03 (0.99, 1.07)	1.08 (1.04, 1.13)
Q4 (18–22)	1.09 (1.06, 1.13)	1.08 (1.04, 1.12)	1.11 (1.07, 1.16)
Q5 (23–45)	1.17 (1.13, 1.21)	1.15 (1.11, 1.19)	1.17 (1.11, 1.22)
Continuous PSI score			
(90^th^ vs 10^th^ percentile)	1.16 (1.12, 1.20)	1.14 (1.10, 1.18)	1.16 (1.11, 1.20)

^*^Model 1: Adjusted for age (continuous, years), sex (male, female), study center (Seoul, Suwon), education (<high school, high school or technical college, ≥university), marital status (unmarried, married, separated, divorced or widowed) and year of visit (2011, 2012, 2013).

^†^Model 2: Further adjusted for smoking (never, former, current), exercise (0, 1–3, >3 times/week) and alcohol (none, moderate).

^‡^Model 3: Further adjusted for body mass index (continuous, kg/m^2^), systolic blood pressure (continuous, mmHg), total and HDL cholesterol (continuous mg/dL), triglycerides (continuous mg/dL), and fasting glucose (continuous mg/dL).

**Figure 2:**
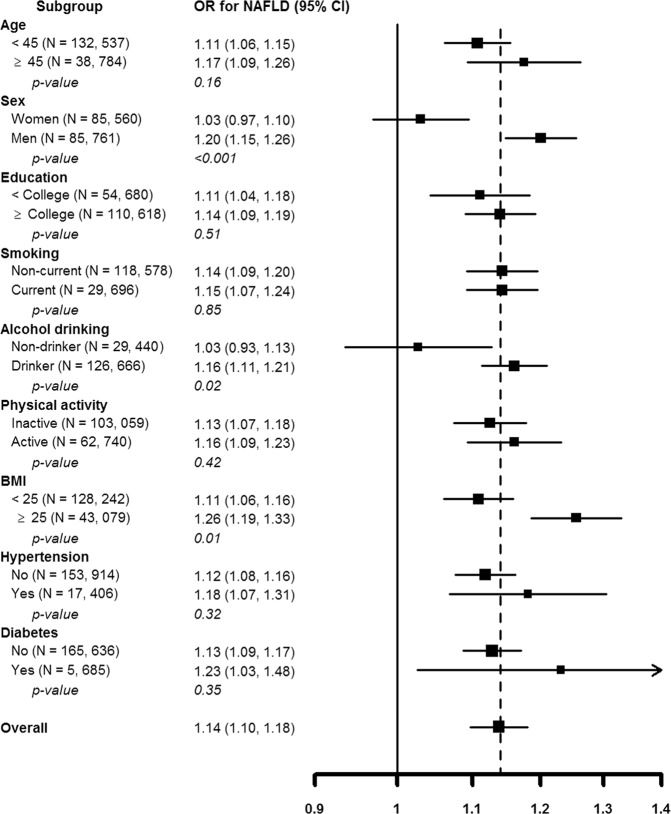
Multivariable-adjusted odds ratios (95% CI) for non-alcoholic fatty liver disease (NAFLD) by PSI stress score. The curves represent adjusted odds ratios (solid line) and their 95% confidence intervals (dashed lines) for NAFLD based on restricted cubic splines for PSI stress score with knots at the 5^th^, 35^th^, 65^th^ and 95^th^ percentiles (PSI scores 9, 13, 18, and 31, respectively) of their sample distributions. The reference value (diamond dot) was set at the 50^th^ percentile (PSI score 15). The model was adjusted for age, sex, study center, education, marital status, year of visit, smoking, vigorous exercise, alcohol, body mass index, systolic blood pressure, total and HDL cholesterol, triglycerides, and fasting glucose.

The positive association between PSI score and NAFLD was observed in all subgroups analyzed (Fig. 2), although the association was stronger in men compared to women (p value for interaction <0.001), and stronger among obese compared to non-obese participants (p value for interaction 0.01).

## Discussion

In this large sample of healthy Korean men and women, perceived stress levels measured as PSI scores were positively associated with the prevalence of NAFLD. The association remained significant even after adjustment for multiple socioeconomic, behavioral, and metabolic factors. To our knowledge, this is the first study to demonstrate a positive association between perceived stress and NAFLD. Our findings support a possible relationship of perceived stress with NAFLD, warranting further research into its underlying mechanism.

The association of perceived stress with cardiovascular and metabolic abnormalities has been evaluated in prior studies^15^. A meta-analysis of longitudinal studies found that overall stress was associated with increasing adiposity^16^. In a prospective study of 6,576 healthy men and women, those with psychological distress as measured using the 12-item version of the General Health Questionnaire, were 1.5 times more likely to have cardiovascular disease than those without stress^4^, and a small prospective cohort study of police officers showed a significant association between stress and metabolic syndrome^17^. Additionally, a recent meta-analysis of individual study participants demonstrated that psychological distress was associated with liver disease mortality which was attributed to 40% of liver-related deaths to probable NAFLD^14^. Our study extends these findings by demonstrating an independent association between perceived stress and NAFLD, the liver manifestation of the metabolic syndrome and one of the most common chronic liver conditions worldwide.

The mechanisms by which stress may contribute to NAFLD are not entirely known. Stress is associated with increased HPA axis activity resulting in production of catecholamines and cortisol, both of which have been associated with NAFLD and insulin resistance^18^. Cortisol, directly or indirectly through insulin-mediated effects on adipose tissue, may induce visceral adiposity^19^ and abnormalities of fatty acid metabolism^20^. Also, in a study of individuals who underwent dexamethasone challenge, 24-hour urinary free cortisol excretion and serum cortisol levels were higher in NAFLD patients than in matched controls, and cortisol levels were closely associated with the severity of liver histology^11^.

Additionally, the association of stress and NAFLD may be mediated by unhealthy behaviors. Indeed, perceived stress has been described as a negative affective state in which individuals engage in unhealthy lifestyles including smoking and alcohol use as coping mechanism^21^. Similar to previous studies^21,22^, we found that study participants with higher stress levels were more likely to current smokers, moderate drinkers, and less likely to perform vigorous exercise than participants with lower stress levels. However, the association between perceived stress levels and NAFLD persisted even after adjusting for smoking, alcohol use and physical activity, suggesting that other pathways are implicated in this association.

The association between perceived stress levels and NAFLD in our study was stronger in men (versus women), and in obese participants (versus non-obese participants). Stress responses can differ by sex. Compared to women, men were more likely to engage in unhealthy lifestyles such as consuming alcohol or smoking^23,24^ or in aggressive strategies^25^ to cope with stress. Moreover, men have greater acute HPA and autonomic responses to standard performance-related psychosocial stressors such as public speaking and arithmetic tasks compared to women^26^. This greater sympathoadrenal responsiveness in men compared to women^27^ may explain the sex-interaction that we observed in our study. Additionally, we also found that the stronger relationship between stress and NAFLD in obese participants. Adipose tissue actively produces and releases adipokines with direct effects on glucose and fat metabolism^28^ as well as interleukin-6 and tumor necrosis factor-alpha which can be involved in NAFLD. It is thus possible that stress is more likely to result in NAFLD in this metabolic milieu. Further mechanistic studies, however, are needed to understand the differences in stress responses in by gender and by obesity levels.

Our study had some limitations. First, we used a cross-sectional design and therefore could not establish temporal relationships or infer causality. However participants were asked to complete the survey questionnaire prior to getting the ultrasonographic results. Second, we used US-detected fatty liver as the study outcome, as histological confirmation or MR spectroscopy^29,30^ are unfeasible in studies of this magnitude. US, however, has an acceptable degree of diagnostic accuracy for steatosis^31^ and is widely used clinically and in population-based studies^29,32^. Furthermore, this type of measurement error is non-differential and likely to result in an underestimation of the association between perceived stress and NAFLD. Third, stress assessment was based on self-report and also subject to measurement error, which would tend to attenuate the observed associations even further. Fourth, the test-retest reliability of PSI score was not available in our study population. We cannot exclude the misclassification of PSI score, NAFLD and other covariates using single measurement. Additionally, we cannot exclude the possibility of some unmeasured or residual confounding in the association between PSI score and prevalent NAFLD. Finally, our results were based on a sample of relatively healthy, young and middle-aged, educated Koreans, and might not be generalizable to other age- and ethnic populations.

Our study also had several strengths. Measurements in the Kangbuk Samsung Cohort Study were obtained by trained personnel using standardized data collection procedures and protocols. We had information on a large number of NAFLD risk factors and were able to adjust for them in multivariable models and, because of the large sample size, we had sufficient power to examine the association across a number of pre-specified clinically relevant subgroups. Additionally, our exposure variable, perceived stress, was measured using a culturally validated tool. Biological markers of stress measure the activation of specific pathways in the stress response^33^, while perceived stress, as measured in our study, also captures information regarding stress appraisal^34^.

In summary, in this large population study of apparently healthy men and women, higher perceived stress was significantly associated with a higher prevalence of NAFLD independent of established risk factors. Our study extends an increasing body of research showing that stress may have adverse metabolic consequences and adds NAFLD to the list of health concerns associated with stress. Prospective studies and interventional trials focused the impact of stress management on metabolic abnormalities and NAFLD are warranted.

## Materials and Methods

### Study population

The Kangbuk Samsung Cohort Study is a cohort study of Korean men and women 18 years of age or older who underwent a comprehensive annual or biennial health examination at the Kangbuk Samsung Hospital Health Screening Centers in Seoul and Suwon, South Korea^35^. Over 80% of participants were employees and their spouses from various companies or local governmental organizations. In South Korea, all employees are required to participate in annual or biennial health screening exams, offered free of charge under the Industrial Safety and Health Law. The remaining participants purchased screening exams out of pocket voluntarily at the health exam center.

**Figure 3:**
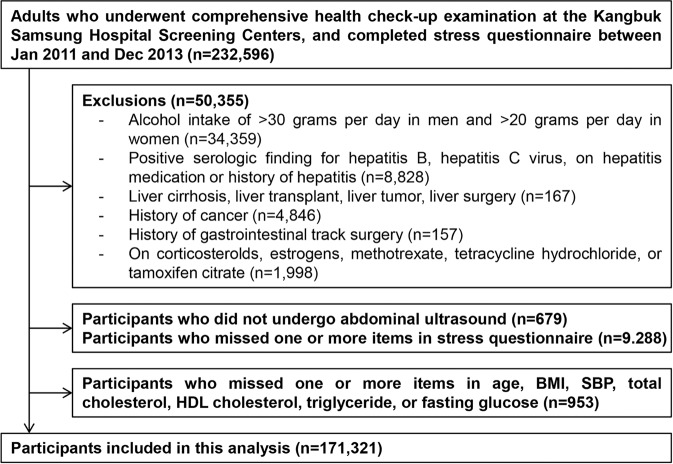
Multivariable-adjusted odds ratios (95% confidence internal) comparing the 90^th^ vs the 10^th^ percentiles of PSI stress score (27 vs. 10) by clinically relevant subgroups. PSI scores were modeled as restricted cubic splines with knots at the 5^th^, 35^th^, 65^th^ and 95^th^ percentiles of the sample distribution. Logistic regression models were adjusted for age, sex, study center, education, marital status, year of visit, smoking, vigorous exercise, alcohol, body mass index, systolic blood pressure, total and HDL cholesterol, triglycerides, and fasting glucose.

The study population for the current analysis consisted of participants who underwent a comprehensive health check-up examination and completed a stress questionnaire between January, 2011 and December, 2013 (n = 232,569; Fig. 3). We excluded participants who had any of the following conditions: excessive alcohol intake, defined as alcohol intake >30 g/d in men or >20 g/d in women (n = 34,359)^36^; self-reported history of hepatitis, use of medications for hepatitis, or serologic evidence of hepatitis B or hepatitis C virus infection (n = 8,828); self-reported history of liver surgery, liver transplant, evidence of liver cirrhosis or liver tumor on US (n = 167); self-reported use of medications that can induce fatty liver, including corticosteroids, estrogens, methotrexate, tetracycline, or tamoxifen (n = 1,997); self-reported history of gastrointestinal track surgery (n = 157); or self-reported history of cancer (n = 4,846). Since participants could meet more than one exclusion criteria, the number of eligible participants was 182,241. We further excluded participants with missing abdominal US (n = 679) or with partially missing data in the stress questionnaires (n = 9,288), with missing covariates of age, BMI, systolic BP, total and HDL cholesterol, triglycerides or fasting glucose (n = 953). The final study sample included 171,321 participants. If a participant had more than one screening exam with a stress questionnaire and US during the study period, only the first exam was included in the analysis.

The Institutional Ethics Committee of the Kangbuk Samsung Hospital approved this study. The Ethics Committee waived the requirement of informed consent because de-identified data routinely collected during health screening visits was used. All methods were carried out in accordance with relevant guidelines and regulations.

### Data collection

Perceived stress was assessed using the short version of the Perceived Stress Inventory (PSI), a validated stress questionnaire developed for the evaluation of perceived stress in the general Korean population by the Korean Center for Disease Control^37^. The questionnaire was developed based on the Stress-induced Cognition Scale^38^, the Stress Response Inventory^39^ and the Perceived Stress Questionnaire^40^. The questionnaire consists of 9 items in 3 domains (tension, depression and anger) and uses a 1–5 Likert response scale. Perceived stress was defined as the total sum of all Likert items in the questionnaire (PSI score), and ranged from 9 to 45 with higher scores indicating increased stress. The test-retest correlation and Cronbach α for internal consistency of the original PSI were 0.73 and 0.91, respectively^37^, and the internal consistency of the PSI in our population was 0.90. PSI scores were categorized in quintiles as there are no clinically established cut-offs.

Abdominal US were performed by eleven experienced radiologists who were all unaware of the study aims^41^. Images were captured in a standard fashion with the patient in the supine position with the right arm raised above the head. An ultrasonographic diagnosis of fatty liver was defined as the presence of a diffuse increase of fine echoes in the liver parenchyma compared with the kidney or spleen parenchyma^42^. The inter- and intra-observer reliability of fatty liver diagnoses were very high (kappa statistics of 0.74 and 0.94, respectively)^41^. Since we had already excluded participants with excessive alcohol use (>20 g/d for women and >30 g/d for men) as well as other identifiable causes of fatty liver at baseline (as described in the exclusion criteria), cases of fatty liver were considered NAFLD^1^.

At the screening visit, demographic characteristics, smoking status, alcohol consumption, physical activity, medical history, and medication use were collected through standardized self-administered questionnaires. Education was categorized into less than high school, high school or technical college, and university or above. Marital status was categorized into unmarried, married, separated, divorced or widowed. Smoking status was categorized into never, former, or current smoker, and alcohol consumption into none or moderate (≤30 grams/day in men and ≤20 grams/day in women). Frequency of moderate- or vigorous-physical activity was categorized into 0, 1 to 3, or >3 times/week.

Height and weight were measured by trained nurses with the participants wearing a light weight hospital gown and no shoes. Body mass index was calculated as weight in kilograms divided by height in meters squared. Blood pressure was measured using an automated Vital Signs Monitor (Model 53000; Welch Allyn, New York, USA) with subjects in the sitting position with the arm supported at the heart level. Three readings were recorded for each individual, and the average of the second and third readings was used for analysis. Hypertension was defined as a systolic blood pressure ≥140 mm Hg, a diastolic blood pressure ≥90 mm Hg, a self-reported history of hypertension, or current use of antihypertensive medications.

Serum total cholesterol, high-density lipoprotein (HDL) cholesterol, triglycerides, and glucose levels in blood samples collected after at least 10 hours of fasting were measured based on previously described methods^35,43^. Diabetes mellitus was defined as a fasting serum glucose ≥126 mg/dL, a self-reported history of diabetes, or current use of antidiabetic medications.

### Statistical analysis

Descriptive statistics were used to summarize the characteristics of participants by the presence of NAFLD. Multivariable logistic regression was used to assess the association between perceived stress levels (PSI score) and NAFLD. For the main analyses, we calculated the multivariable-adjusted odds ratios (OR) and 95% confidence intervals (CI) of NAFLD prevalence comparing the four highest quintiles of PSI score to the lowest quintile. In addition to categorical analysis, we modeled PSI scores as continuous variables using restricted cubic splines with knots at the 5^th^, 35^th^, 65^th^ and 95^th^ percentiles of the sample distribution to provide a flexible estimate of the dose-response relationship between PSI score and NAFLD.

We used three models with increasing degrees of adjustment to account for potential confounding factors and to evaluate the role of potential biological mediators. In our study, we defined confounding variables using the following criteria: Each variable is 1) causally associated with the outcome (NAFLD) and 2) non-causally or causally associated with the exposure (PSI score) but 3) is not an intermediate variable in the causal pathway between exposure (PSI score) and the outcome (NAFLD). The first model adjusted for age, sex, study center, education, marital status, and year of visit. The second model was further adjusted for smoking, exercise and alcohol consumption. The third model was further adjusted for potential biological mediators and metabolic risk factors: body mass index, systolic blood pressure, total and HDL cholesterol, triglycerides, and fasting glucose. Missing categorical covariates were set as additional categories and included in the models.

To assess the heterogeneity of associations between PSI score and prevalent NAFLD, additional analyses were performed by pre-specified clinically relevant subgroups defined by age (<45 vs. ≥45 years), sex (women vs. men), education, smoking (current vs. noncurrent smokers), alcohol drinking (non-drinker vs. moderate drinker), physical activity (<3 vs. ≥3 times/week), body mass index (<25 vs. ≥25 kg/m^2^), hypertension (yes vs. no), and diabetes (yes vs. no). We tested for the interaction of PSI scores with clinical characteristics using Wald tests for cross-product terms in regression models. All reported p values were two-sided and the significance level was set at 0.05. All analyses were performed using STATA version 12 (StataCorp LP, College Station, TX, USA).

## Supplementary information

Supplementary table.

## Data Availability

The data, and study materials will not be made available to other researchers for purposes of reproducing the results. However, analytical methods are available from corresponding author on reasonable request.
