# Author Correction: Perceived stress and non-alcoholic fatty liver disease in apparently healthy men and women

**DOI:** 10.1038/s41598-020-78911-0

**Published:** 2020-12-09

**Authors:** Danbee Kang, Di Zhao, Seungho Ryu, Eliseo Guallar, Juhee Cho, Mariana Lazo, Hocheol Shin, Yoosoo Chang, Eunju Sung

**Affiliations:** 1 Department of Clinical Research Design & Evaluation, SAIHST, Sungkyunkwan University, Seoul, Korea; 2 Center for Clinical Epidemiology, Samsung Medical Center, Sungkyunkwan University School of Medicine, Seoul, Korea; 3 Department of Epidemiology and Welch Center for Prevention, Epidemiology, and Clinical Research, Johns Hopkins University Bloomberg School of Public Health, Baltimore, Maryland United States of America; 4 Center for Cohort Studies, Total Healthcare Center, Kangbuk Samsung Hospital, Sungkyunkwan University School of Medicine, Seoul, Korea; 5 Department of Occupational and Environmental Medicine, Kangbuk Samsung Hospital, Sungkyunkwan University School of Medicine, Seoul, Korea; 6 Department of the Family Medicine, Kangbuk Samsung Hospital, Sungkyunkwan University School of Medicine, Seoul, Korea

Correction to: *Scientific Reports* 10.1038/s41598-019-57036-z, published online 8 January 2020

This Article contains an error in the Figure legends of Figure 1, Figure 2 and Figure 3. The legends of these Figures were inadvertently switched.

The legend of Figure 1:

“Flowchart of study participants.”

should read:

“Multivariable-adjusted odds ratios (95% CI) for non-alcoholic fatty liver disease (NAFLD) by PSI stress score. The curves represent adjusted odds ratios (solid line) and their 95% confidence intervals (dashed lines) for NAFLD based on restricted cubic splines for PSI stress score with knots at the 5^th^, 35^th^, 65^th^ and 95^th^ percentiles (PSI scores 9, 13, 18, and 31, respectively) of their sample distributions. The reference value (diamond dot) was set at the 50^th^ percentile (PSI score 15). The model was adjusted for age, sex, study center, education, marital status, year of visit, smoking, vigorous exercise, alcohol, body mass index, systolic blood pressure, total and HDL cholesterol, triglycerides, and fasting glucose.”

The legend of Figure 2:

“Multivariable-adjusted odds ratios (95% CI) for non-alcoholic fatty liver disease (NAFLD) by PSI stress score. The curves represent adjusted odds ratios (solid line) and their 95% confidence intervals (dashed lines) for NAFLD based on restricted cubic splines for PSI stress score with knots at the 5^th^, 35^th^, 65^th^ and 95^th^ percentiles (PSI scores 9, 13, 18, and 31, respectively) of their sample distributions. The reference value (diamond dot) was set at the 50^th^ percentile (PSI score 15). The model was adjusted for age, sex, study center, education, marital status, year of visit, smoking, vigorous exercise, alcohol, body mass index, systolic blood pressure, total and HDL cholesterol, triglycerides, and fasting glucose.”

should read:

“Multivariable-adjusted odds ratios (95% confidence interval) comparing the 90^th^ vs the 10^th^ percentiles of PSI stress score (27 vs. 10) by clinically relevant subgroups. Logistic regression models were adjusted for age, sex, study center, education, marital status, year of visit, smoking, vigorous exercise, alcohol, body mass index, systolic blood pressure, total and HDL cholesterol, triglycerides, and fasting glucose.”

The legend of Figure 3:

“Multivariable-adjusted odds ratios (95% confidence internal) comparing the 90^th^ vs the 10^th^ percentiles of PSI stress score (27 vs. 10) by clinically relevant subgroups. PSI scores were modeled as restricted cubic splines with knots at the 5^th^, 35^th^, 65^th^ and 95^th^ percentiles of the sample distribution. Logistic regression models were adjusted for age, sex, study center, education, marital status, year of visit, smoking, vigorous exercise, alcohol, body mass index, systolic blood pressure, total and HDL cholesterol, triglycerides, and fasting glucose.”

should read:

“Flowchart of study participants.”

