# Supplementary material for: Perceived stress and non-alcoholic fatty liver disease in apparently healthy men and women
Source: Sci Rep. 2020 Jan 8;10:38. doi: 10.1038/s41598-019-57036-z (PMC6949298; doi:10.1038/s41598-019-57036-z)
Supplement: Supplementary file 1 — Supplementary table. [file 41598_2019_57036_MOESM1_ESM.docx]

**Supplemental Material**

**Perceived stress and non-alcoholic fatty liver disease in apparently healthy men and women**

Danbee Kang^1,2,†^, Di Zhao^3,†^, Seungho Ryu^1,4,5^, Eliseo Guallar^3^, Juhee Cho^1-4^, Mariana Lazo^3^, Hocheol Shin^6^, Yoosoo Chang^1,4,5,*^, Eunju Sung^6,*^

^1^Department of Clinical Research Design & Evaluation, SAIHST, Sungkyunkwan University, Seoul, Korea

^2^Center for Clinical Epidemiology, Samsung Medical Center, Sungkyunkwan University School of Medicine, Seoul, Korea

^3^Department of Epidemiology and Welch Center for Prevention, Epidemiology, and Clinical Research, Johns Hopkins University Bloomberg School of Public Health, Baltimore, Maryland, United States of America

^4^Center for Cohort Studies, Total Healthcare Center, Kangbuk Samsung Hospital, Sungkyunkwan University School of Medicine, Seoul, Korea

^5^Department of Occupational and Environmental Medicine, Kangbuk Samsung Hospital, Sungkyunkwan University, School of Medicine, Seoul, Korea

^6^Department of the Family Medicine, Kangbuk Samsung Hospital, Sungkyunkwan University School of Medicine, Seoul, Korea

**^†^Contributed equally as co-first authors of the manuscript.**

**^*^Corresppondences**

Eunju Sung, Department of Family Medicine, Kangbuk Samsung Hospital, Sungkyunkwan University School of Medicine, 29 Saemunan-ro, Jongno-gu, Seoul 03181, Korea.

Tel: +82-2-2001-2277; Fax: 82-2-757-0436; E-mail: [eunjusung68@gmail.com](mailto:eunjusung68@gmail.com)

or

Yoosoo Chang, Department of Occupational and Environmental Medicine, Sungkyunkwan University School of Medicine, Samsung Main Building B2, 250 Taepyung-ro 2ga, Jung-gu, Seoul 04514, Korea.

Tel: 82-2-2001-5139; Fax: 82-2-757-0436; E-mail: [yoosoo.chang@gmail.com](mailto:yoosoo.chang@gmail.com)

| **Supplement Table 1.** Baseline characteristics of study participants and by PSI score quintiles^*^ | | | | | | |
| --- | --- | --- | --- | --- | --- | --- |
| Characteristics | PSI scores | | | | | p value |
|  | 1^st^ quintile (9-11) | 2^nd^ quintile (12-14) | 3^rd^ quintile (15-17) | 4^th^ quintile (18-22) | 5^th^ quintile (23-45) |  |
| Age (years) | 41.8 (10.2) | 40.2 (9.0) | 39.6 (8.5) | 38.8 (8.2) | 37.8 (8.0) | <0.001 |
| Sex |  |  |  |  |  | <0.001 |
| Female | 18,136 (47.5) | 18,050 (50.0) | 16,980 (49.9) | 15,844 (48.7) | 16,550 (54.3) |  |
| Male | 20,038 (52.5) | 18,043 (50.0) | 17,053 (50.1) | 16,675 (51.3) | 13,952 (45.7) |  |
| Education |  |  |  |  |  | <0.001 |
| <High school | 1,686 (4.4) | 955 (2.6) | 795 (2.3) | 660 (2.0) | 661 (2.2) |  |
| High school or technical college | 11,791 (30.9) | 10,308 (28.6) | 9,665 (28.4) | 9,174 (28.2) | 8,985 (29.5) |  |
| ≥University | 23,357 (61.2) | 23,603 (65.4) | 22,341 (65.6) | 21,547 (66.3) | 19,770 (64.8) |  |
| Marital status |  |  |  |  |  | <0.001 |
| Unmarried | 4,420 (11.6) | 4,798 (13.3) | 4,864 (14.3) | 5,477 (16.8) | 5,934 (19.5) |  |
| Married | 32,194 (84.3) | 30,076 (83.3) | 28,033 (82.4) | 25,981 (79.9) | 23,431 (76.8) |  |
| Separated, divorced or widowed | 907 (2.4) | 678 (1.9) | 609 (1.8) | 547 (1.7) | 630 (2.1) |  |
| Study center |  |  |  |  |  | <0.001 |
| Seoul | 23,872 (62.5) | 22,754 (63.0) | 21,274 (62.5) | 20,188 (62.1) | 18,834 (61.7) |  |
| Suwon | 14,302 (37.5) | 13,339 (37.0) | 12,759 (37.5) | 12,331 (37.9) | 11,668 (38.3) |  |
| NAFLD | 10,983 (28.8) | 9,868 (27.3) | 9,334 (27.4) | 9,118 (28.0) | 8,235 (27.0) |  |
| Smoking |  |  |  |  |  | <0.001 |
| Never | 20,742 (54.3) | 20,199 (56.0) | 18,697 (54.9) | 17,789 (54.7) | 17,422 (57.1) |  |
| Former | 5,837 (15.3) | 5,088 (14.1) | 4,645 (13.6) | 4,429 (13.6) | 3,730 (12.2) |  |
| Current | 5,664 (14.8) | 5,780 (16.0) | 6,157 (18.1) | 6,284 (19.3) | 5,811 (19.1) |  |
| Alcohol |  |  |  |  |  | <0.001 |
| None | 7,279 (19.1) | 6,351 (17.6) | 5,844 (17.2) | 5,188 (16.0) | 4,778 (15.7) |  |
| Moderate | 26,705 (70.0) | 26,420 (73.2) | 25,219 (74.1) | 24,891 (76.5) | 23,431 (76.8) |  |
| Vigorous exercise (times/week) |  |  |  |  |  | <0.001 |
| 0 | 21,780 (57.1) | 21,298 (59.0) | 20,657 (60.7) | 19,681 (60.5) | 19,643 (64.4) |  |
| 1-3 | 11,578 (30.3) | 11,104 (30.8) | 10,253 (30.1) | 10,061 (30.9) | 8,332 (27.3) |  |
| >3 | 3,215 (8.4) | 2,487 (6.9) | 2,068 (6.1) | 1,870 (5.8) | 1,772 (5.8) |  |
| Body mass index (kg/m^2^) | 23.2 (3.2) | 23.0 (3.2) | 22.9 (3.2) | 23.0 (3.3) | 22.9 (3.5) | <0.001 |
| Total cholesterol (mg/dL) | 195.4 (34.3) | 194.1 (34.1) | 193.8 (34.1) | 193.5 (34.1) | 192.1 (33.7) | <0.001 |
| HDL cholesterol (mg/dL) | 57.9 (14.7) | 58.2 (14.7) | 58.2 (14.6) | 58.1 (14.7) | 58.7 (14.8) | <0.001 |
| Triglycerides (mg/dL)^†^ | 90.0 (64.0 - 132.0) | 87.0 (62.0 - 129.0) | 87.0 (62.0 - 129.0) | 88.0 (62.0 - 130.0) | 85.0 (61.0 - 127.0) | <0.001 |
| Systolic blood pressure (mmHg) | 109.6 (13.6) | 108.3 (13.3) | 107.9 (13.1) | 107.6 (13.0) | 106.4 (12.7) | <0.001 |
| Hypertension | 4,767 (12.5) | 3,707 (10.3) | 3,374 (9.9) | 3,068 (9.4) | 2,490 (8.2) | <0.001 |
| Diabetes | 1,579 (4.1) | 1,185 (3.3) | 1,056 (3.1) | 998 (3.1) | 867 (2.8) | <0.001 |
| ^*^Values are means (SD) or number (%).  ^†^Values are median (IQR)  NAFLD = Nonalcoholic fatty liver disease; PSI = Perceive Stress Inventory | | | | | | |

| **Supplementary Table 2.** Crude and adjusted prevalence (standard error) of NAFLD by PSI score quintiles | | | | | | |
| --- | --- | --- | --- | --- | --- | --- |
|  | PSI scores | | | | | p value |
|  | 1^st^ quintile  (9-11) | 2^nd^ quintile  (12-14) | 3^rd^ quintile  (15-17) | 4^th^ quintile  (18-22) | 5^th^ quintile  (23-45) |  |
| Crude | 28.8 (0.2) | 27.3 (0.2) | 27.4 (0.2) | 28.0 (0.2) | 27.0 (0.3) | <0.001 |
| Age, and sex -adjusted | 26.7 (0.2) | 27.1 (0.2) | 27.5 (0.2) | 28.3 (0.2) | 29.5 (0.3) | <0.001 |
| Age, sex and BMI-adjusted | 26.6 (0.2) | 27.4 (0.2) | 28.0 (0.2) | 28.3 (0.2) | 28.9 (0.2) | <0.001 |

Data are crude and adjusted prevalence (standard error).

BMI = Body mass index; NAFLD = Nonalcoholic fatty liver disease; PSI = Perceive Stress Inventory
